# Dopamine internalization via Uptake_2_ and stimulation of intracellular D_5_-receptor-dependent calcium mobilization and CDP-diacylglycerol signaling

**DOI:** 10.3389/fphar.2024.1422998

**Published:** 2024-10-25

**Authors:** Wenfei Kang, Arlette Deukam Siewe, Chizurum C. Oluigbo, Mercy O. Arijesudade, Eugen Brailoiu, Ashiwel S. Undieh

**Affiliations:** ^1^ Department of Biomedical Sciences, School of Medicine, City University of New York, New York, NY, United States; ^2^ Neuroscience Collaborative, The Graduate Center, City University of New York, New York, NY, United States; ^3^ Department of Pharmacology, School of Medicine, Temple University, Philadelphia, PA, United States

**Keywords:** dopamine uptake, CDP-diacylglycerol, nucleolipid, phosphatidylinositol, calcium mobilization, D5 dopamine receptor, uptake2, plasmamembrane monoamine transporter

## Abstract

Dopamine stimulates CDP-diacylglycerol biosynthesis through D_1_-like receptors, particularly the D_5_ subtype most of which is intracellularly localized. CDP-diacylglycerol regulates phosphatidylinositol-4,5-bisphosphate-dependent signaling cascades by serving as obligatory substrate for phosphatidylinositol biosynthesis. Here, we used acute and organotypic brain tissues and cultured cells to explore the mechanism by which extracellular dopamine acts to modulate intracellular CDP-diacylglycerol. Dopamine stimulated CDP-diacylglycerol in organotypic and neural cells lacking the presynaptic dopamine transporter, and this action was selectively mimicked by D_1_-like receptor agonists SKF38393 and SKF83959. Dopaminergic CDP-diacylglycerol stimulation was blocked by decynium-22 which blocks Uptake_2_-like transporters and by anti-microtubule disrupters of cytoskeletal transport, suggesting transmembrane uptake and guided transport of the ligands to intracellular sites of CDP-diacylglycerol regulation. Fluorescent or radiolabeled dopamine was saturably transported into primary neurons or B35 neuroblastoma cells expressing the plasmamembrane monoamine transporter, PMAT. Microinjection of 10-nM final concentration of dopamine into human D_5_-receptor-transfected U2-OS cells rapidly and transiently increased cytosolic calcium concentrations by 316%, whereas non-D_5_-receptor-expressing U2-OS cells showed no response. Given that U2-OS cells natively express PMAT, bath application of 10 μM dopamine slowly increased cytosolic calcium in D_5_-expressing cells. These observations indicate that dopamine is actively transported by a PMAT-implicated Uptake_2_-like mechanism into postsynaptic-type dopaminoceptive cells where the monoamine stimulates its intracellular D_5_-type receptors to mobilize cytosolic calcium and promote CDP-diacylglycerol biosynthesis. This is probably the first demonstration of functional intracellular dopamine receptor coupling in neural tissue, thus challenging the conventional paradigm that postsynaptic dopamine uptake serves merely as a mechanism for deactivating spent or excessive synaptic transmitter.

## 1 Introduction

CDP-diacylglycerol (cytidine diphosphate diacylglycerol) is a nucleolipid synthesized in eukaryotic cells from cytidine triphosphate and selective species of phosphatidic acid (Heacock and Agranoff, 1997; Lykidis et al., 1997; Monaco and Feldman, 1997; Undieh, 2010). CDP-diacylglycerol biosynthesis in the endoplasmic reticulum (ER) is catalyzed by CDP-diacylglycerol synthase (CDS) of which two vertebrate isoforms–*cds1* and *cds2* – are expressed (Halford et al., 1998; Inglis-Broadgate et al., 2005; Mercade et al., 2007). The subsequent reaction of CDP-diacylglycerol with *myo*-inositol, catalyzed by phosphatidylinositol synthase, yields phosphatidylinositol (PI) (Imai and Gershengorn, 1987; Antonsson, 1997; Lykidis et al., 1997). The latter is transported by carrier proteins from intracellular organelles, chiefly the ER, to the cell membrane where it is sequentially phosphorylated to phosphatidylinositol-4,5-bisphosphate (PIP_2_) (Alb et al., 1996; Lete et al., 2020). PIP_2_ regulates critical cellular processes in serving as a ubiquitous membrane anchor and as an obligatory substrate for multi-functional phospholipase C and phosphatidylinositol-3-kinase signaling cascades (Shah et al., 2013; De Craene et al., 2017; Hammond and Burke, 2020; Mandal, 2020).

Dopamine has been reported to promote PIP2 metabolism through activation of D_1_-like (D_1_/D_5_) receptors. (Felder et al., 1989; Mahan et al., 1990; Undie and Friedman, 1990; Undie et al., 1994). Dopamine and D_1_-like agonists have also been shown to increase the biosynthesis of CDP-diacylglycerol in tissues that are associated with dopaminergic stimulation of PIP2 metabolism (Undie, 1999; Sahu et al., 2009). Evidence suggests that CDP-diacylglycerol induced by dopamine agonists in brain tissue is converted to phosphatidylinositides which are then available to enhance subsequent responses to stimulation of phospholipase C-coupled receptors such as muscarinic M_3_ and serotonin 5HT_2_ receptors (Tyeryar and Undie, 2007). Selective receptor knockout studies have suggested a major involvement of the D_5_-type receptor in dopaminergic modulation of CDP-diacylglycerol biosynthesis (Sahu et al., 2009). Subcellular fractionation studies revealed that the D_5_ receptor is distributed predominantly within the intracellular compartment, probably anchored to organellar bilaminar membranes, in forebrain regions expressing the receptor, and that only a minor component of total receptor expression is associated with the cellular plasmamembrane (Voulalas et al., 2011). If the plasmamembrane contingent of D_5_ receptors were not sufficient to account for the CDP-diacylglycerol effect, then a strategy must exist for extracellular dopamine to modulate intracellular D_5_ receptors and to promote microsomal or mitochondrial CDP-diacylglycerol biosynthesis.

Toward examining potential mechanisms that may contribute to dopaminergic modulation of intracellular CDP-diacylglycerol biosynthesis, we hypothesized that extracellular dopamine enters into neural cells where it interacts with intracellular D_5_ receptors to initiate signaling events that ultimately lead to enhanced formation of CDP-diacylglycerol. Depending on the cell type, active transport of dopamine into cells could be mediated by the presynaptic dopamine transporter, DAT (*SLC6A3*) (Giros et al., 1992), or by the postsynaptic monoamine transporter, Uptake2 (Page et al., 1998; Wang, 2016; Gasser, 2021). The term “postsynaptic” in this context, refers to Uptake_2_-bearing dopaminoceptive cells as opposed to presynaptic DAT-expressing dopaminergic neurons. Given that DAT is distributed at presynaptic terminals in the forebrain, its role can be estimated or discounted by use of *in vitro* tissue preparations that are devoid of presynaptic terminals. For purposes of postsynaptic dopamine transport, classical Uptake2 is now known to consist of the plasmamembrane monoamine transporter (PMAT or ENT4, *SLC29A4*) (Engel et al., 2004; Dahlin et al., 2007; Vialou et al., 2007; Duan and Wang, 2010), and isoforms of organic cation transporters, principally OCT3 (*SLC22A3*) (Vieira and Wang, 2021). PMAT is highly expressed in plasma membranes of neurons at various dopaminoceptive brain regions (Engel et al., 2004; Dahlin et al., 2007; Miura et al., 2017). The rodent and human forms of this low-affinity high-capacity transporter show functional similarities in saturable dopamine transport, with Km values of 201 μM, 271 μM and 466 µM for the human, rat, and mouse variants, respectively (Miura et al., 2017; Shirasaka et al., 2017).

In the present work we queried whether dopamine is actively transported by Uptake_2_ into postsynaptic-type cells where the transmitter might then interact with intracellularly localized D_5_ receptors to lead onward to CDP-diacylglycerol biosynthesis. We used complementary experimental strategies taking advantage of the unique features of organotypic brain slice preparations as well as primary neurons and clonal cell lines. These features included the absence of the presynaptic dopamine terminals and DAT (organotypic slices, primary neurons, B35 neuroblastoma cells), clonal cell expressing the dopamine-preferring Uptake_2_-like transporter PMAT (B35 cells, U2OS osteosarcoma cells), clonal cell expressing or not expressing the CDP-diacylglycerol-associated D_5_ dopamine receptor (B35 cells but *not* U2OS cells), and large cell Soma for intracellular microinjection (U2OS cells). Our observations indicate that dopaminergic activation of CDP-diacylglycerol is a postsynaptic-type response executed by dopamine receptor-expressing cells, and probably involves: (i) uptake of dopamine into the postsynaptic neuron via a PMAT-mediated Uptake_2_-like mechanism, (ii) cytosolic dopamine activation of intracellular D_5_ receptors accompanied with increases in intracellular calcium mobilization, and (iii) induction of microsomal CDP-diacylglycerol biosynthesis which may then lead to onward conversion of the nucleolipid to PI and membrane PIP2. Thus, besides its role in terminating synaptic transmitter action, Uptake_2_/PMAT-mediated postsynaptic intracellular dopamine uptake may serve toward modulating physiologic CDP-diacylglycerol biosynthesis and thereby PI-dependent signaling cascades.

## 2 Materials and methods

### 2.1 Materials and assay systems

#### 2.1.1 Drugs and reagents

SKF38393 (1-Phenyl-2,3,4,5-tetrahydro-(1H)-3-benzazepine-7,8-diol hydrochloride) and SKF83959 (3-methyl-6-chloro-7,8-hydroxy-1-(3-methylphenyl)-2,3,4,5-tetrahydro-1H-3-benzazepine) were obtained from the National Institute of Mental Health Chemical Synthesis Program (NIMH, Bethesda, MD). Dopamine and other pharmacological agents were purchased from SigmaAldrich (sigmaaldrich.com) or Tocris (tocris.com). The radionuclides 5-[^3^H]inositol, 5-[^3^H]cytidine [^14^C]cytidine, and [^3^H]dopamine, were purchased from American Radiolabeled Chemicals (ARCinc.com). All other reagents were procured from Fisher Scientific (Fishersci.com), ThermoFisher Scientific (ThermoFisher.com) or other commercial vendors as indicated. Unless otherwise indicated, drugs were dissolved in assay buffer or in tissue culture-grade double-deionized water (DDW) and then diluted into assay buffer to obtain desired use concentrations. Decynium-22 was dissolved in dimethylsulfoxide (DMSO) while corticosterone was dissolved in ethanol and each stock solution was then diluted in reaction buffer to use concentrations. Each experiment was performed on multiple occasions, each time using fresh preparations of drugs and test tissues. Protein was assayed by the Bradford method using Biorad reagents (Biorad.com) or by Nanodrop spectrophotometry (ThermoFisher.com).

#### 2.1.2 Animals

Male and female Sprague-Dawley rats weighing 225–275 g or timed pregnant dams were sourced from Taconic Farms (Germantown, NY). Animals were housed in climate-controlled facilities with a 12-h light/dark cycle and allowed free access to food and water. Protocols for the care and use of research animals, including the methods of euthanasia, were approved by the Institutional Animal Care and Use Committee (IACUC Protocol #1095) and conformed to the principles set forth in the National Institutes of Health Guide for the Care and Use of Laboratory Animals.

#### 2.1.3 Cell lines

Rat embryonic cortex-derived B35 neuroblastoma cells were purchased and authenticated from mycoplasma-free stock of American Type Culture Collection (ATCC.org) and maintained in DMEM media (Life Technologies/ThermoFisher.com) supplemented with 10% fetal bovine serum, FBS (Atlanta Biological, bio-techne.com) under an atmosphere of 5% CO_2_ and 37°C. DMEM media was switched to Neurobasal serum-free media at least 24 h before cells were used in experiments.

Human U2-OS osteosarcoma cells obtained with authentication from mycoplasma-free stock of ATCC.org were cultured in DMEM containing 10% FBS under 5% CO_2_ and 37°C.

### 2.2 Experimental procedures

#### 2.2.1 Measurement of [^3^H]CDP-diacylglycerol formation in acute brain slice preparations

Agonist-induced accumulation of CDP-diacylglycerol was measured in brain slice preparations according to previously described methods with some modifications (Undie, 1999; Sahu et al., 2009). Briefly, male or female rats were rapidly decapitated and the brains removed and rinsed in calcium-free HEPES bicarbonate (HB) buffer at room temperature (∼23°C) (Undie and Friedman, 1990). Brain regions of interest were dissected out at room temperature and 350 × 350 µm slices were prepared using a McIlwain tissue chopper. Slices were quickly washed several times with room-temperature calcium-free HB buffer before preincubation in 4 volumes of buffer at 37°C for 45 min (1 volume ≈1 mL per 50 mg tissue). Slices were washed once with 4 volumes of normal HB buffer (containing 1.2 mM CaCl_2_) that had been prewarmed to 37°C; the wash buffer was removed by aspiration to obtain a slurry of packed slices. While continually swirling the tube to prevent the slices from sitting packed, 25-µL aliquots of the slices (200–250 µg protein) were distributed into 5-mL polypropylene tubes containing 125 µL of normal HB buffer. Tubes were incubated at 37°C in a Dubnoff water bath with continuous gentle shaking to keep the slices dispersed. After 10 min, 0.6 µCi of 5-[^3^H]cytidine (30 Ci/mmol) was added to each sample and mixed, followed by addition of 5 mM LiCl. Drugs were added 5–10 min after LiCl, the final reaction volume was adjusted to 250 μL, and incubation continued for a further 60 min or as indicated. Reactions were stopped by mixing the slices with 1.5 mL of chloroform-methanol-1M HCl (100:200:1). The mixture was continually vortexed for 30 min at room temperature, 0.5 mL chloroform was added and mixed, followed by 0.75 mL DDW. After vigorous vortexing, samples were centrifuged at 3,000 *g* for 5 min in order to separate the organic and aqueous phases of the extract. Using a positive displacement pipet, a 500-µL aliquot of the lower organic phase was quantitatively transferred into scintillation vials. The samples were allowed to dry under the fume hood, 5 mL of Biosafe scintillation cocktail was added, tubes were vigorously vortexed, and the radioactivity measured by liquid scintillation counting with preprogrammed conversion of counts to disintegrations per minute (DPM). Having used [^3^H]cytidine as the radionuclide, measured radioactivity from the lower organic phase would correspond to [^3^H]CDP-diacylglycerol with negligible contamination from any other radiolabeled lipophilic derivatives of [^3^H]cytidine.

#### 2.2.2 Measurement of [^3^H]CDP-diacylglycerol formation in organotypic striatal slice cultures

The organotypic slice culture method was modified from that of Stoppini and colleagues (Stoppini et al., 1991). Brains were removed from anesthetized neonatal (P0-P1) rat pups of mixed sex and the striata were quickly dissected out and washed in cold Hank’s Balanced Salts Solution (HBSS, Life Technologies/ThermoFisher.com). Dissected tissues were cut into 400 μm-thick coronal sections using an Oscillating Tissue Slicer (Electron Microscopy Sciences, EmsDiasum.com). The slices were placed atop Millicell 0.4 μm microporous transparent biopore membranes (Millicell-CM, 12 mm, Millipore.com). Two slices were placed onto one insert and the insert placed into individual wells of a 24-well microplate. Wells were supplied with 400 μL Neurobasal medium (GIBCO/ThermoFisher #12348017) supplemented with modified B27 from which antioxidants had been removed (GIBCO/ThermoFisher #10889038). Cultures were incubated at 37°C under an atmosphere of 5% CO_2_ and 95% air with media change the day after dissection and thereafter every 3 days.

To determine endogenous dopamine levels in the *in vitro* tissues, dopamine radioimmunoassays were performed on samples of organotypic cultured tissues after 0, 1, 2, 3 and 7 days in culture. For this, organotypic tissue samples were extracted by sonication in 0.1 M HCl (≈10 mg tissue/mL) followed by centrifugation at 10,000 x *g* for 5 min. The clear supernatant was tested for dopamine content using the dopamine [^125^I]-RIA kit (ALPCO.com, #017-RA604/50) following the manufacturer’s recommended procedures.

The CDP-diacylglycerol assay was conducted on organotypic rat striatal slices maintained *in vitro* for 7 days essentially as described (Murphy et al., 1992; Undie, 1999). Organotypic slices were incubated with fresh Neurobasal culture media containing 3 μCi [^3^H]cytidine in 400 µL media which was distributed 320 μL in the well (underneath the insert) and 80 μL added over the tissue and the overflow allowed to drain through the Millicell insert. After 30 min, 50 μL of 10 mM LiCl was added (40 μL in the well and 10 μL in the insert) to yield a final Li + concentration of 1 mM. After 15 min, 50 μL of drug solutions (at 10X final concentration) were added directly into the inserts and then 50 µL of media was added into the insert to bring the final reaction volume to 500 µL. Slices were incubated under an atmosphere of 5% CO_2_- 95% air for 60 min and the reaction was terminated by immersing the inserts with the slices into ice-cold HB buffer. The slices were washed off the membrane with a bottom-up jet of cold buffer and then transferred into 5-mL polypropylene centrifuge tubes. Following a quick spin, the slices were resuspended in 250 µL DDW, 1.5 mL of chloroform-methanol-1M HCl (100:200:1) was added, and the mixture processed for extraction and quantification of radiolabeled CDP-diacylglycerol as described above for acute brain slices.

#### 2.2.3 CDP-diacylglycerol formation in B35 neuroblastoma cells

Rat B35 cells were re-plated into 24-well plates at a seeding density of 5 × 10^4^ and grown to 80% confluence (∼5 × 10^5^ cells). Cells were induced to differentiate by reducing the serum content to 1% and adding dibutyryl cyclic-AMP 0.5 mM. Cells were then used for assay after 3 days. Differentiated B35 cells were incubated in 400 µL of complete Neurobasal medium at 37°C for 3 h. Subsequent treatments were added at 20X final concentrations in 25-µL volumes. Cells were labeled with tritiated cytidine 0.3 µCi/well (specific activity 30 Ci/mmol) for 30 min. LiCl 1 mM final concentration was added followed after 5 min by addition of test agents. The final incubation volume was adjusted to 500 µL. Incubation was continued at 37°C for 60 min and the reaction was stopped by transferring the plate into an ice bath and adding 500 µL of ice-cold PBS. The cells were detached by scraping with a rubber policeman, collected by centrifugation, resuspended in 250 µL cold DDW in a 5-mL polypropylene tube, and 1.5 mL chloroform/methanol/1M HCl (100:200:1) added. The mixture was processed for extraction and measurement of CDP-diacylglycerol radioactivity as described above for acute brain slices.

#### 2.2.4 CDP-diacylglycerol biosynthesis and dopamine uptake in primary cortical neurons

Rat frontal cortex neurons were prepared from neonatal (P0-P1) rat pups using the Miltenyi GentleMACS Octo Dissociator in accordance with the manufacturer’s recommended reagents and procedures. Dissociated neurons were plated at a density of 3 × 10^5^ cells per well of a 24-well plate in 500 µL Neurobasal medium (ThermoFisher Neurobasal-plus #A3582901, B27-plus #A3582801, glutamax #35050061, penicillin/streptomycin #15140148, reconstituted according to manufacturer recommendations) with media change after 24 h and every 3 days thereafter. Cells were used after 7 days.

To measure CDP-diacylglycerol accumulation, dissociated cortical cells were plated at a density of 6 × 10^5^ cells per well in 12-well plates and grown in 1,000 µL Neurobasal medium at 37°C with media change every 2–3 days. After 5–7 days, culture media was changed to media containing tritiated cytidine 0.3 µCi/well (specific activity 30 Ci/mmol) and the cells incubated for 30 min. Dopamine and other test agents were added and the final incubation volume was adjusted to 1,000 µL. LiCl was omitted in these cell-based assays seeing that the cation did not appreciably improve CDP-diacylglycerol accumulation in this system. Incubation was continued at 37°C for 60 min and the reaction was stopped by transferring the plate into an ice bath and adding 500 µL of ice-cold PBS. The cells were detached by scraping with a rubber policeman, collected by centrifugation, resuspended in 250 µL cold DDW in a 5-mL polypropylene tube, and 1.5 mL chloroform/methanol/1M HCl (100:200:1) added. The mixture was processed for extraction and measurement of CDP-diacylglycerol radioactivity as described above for acute brain slices.

To measure [^3^H]dopamine uptake, the culture media was replaced with 400 µL of fresh Neurobasal media and incubated for 30 min, then indicated concentrations of decynium-22 or other treatment agent in Neurobasal media was added. After 5 min, 100 µM or other indicated concentration of [^3^H]dopamine (prepared as described below for B35 cells) was added and the final reaction volume adjusted to 500 µL using fresh Neurobasal media. Plates were incubated at 37°C for 30 min, transferred to an ice bath, and 500 µL of cold PBS quickly added to each well to stop the reaction. Cells were washed five times with 500 µL volumes of ice-cold PBS, then another 400 µL of cold PBS was added and the cells detached by scraping. The mixture from each well was transferred to a 2-mL Eppendorf tube, 100-µL rinsings of the wells was added to respective tubes, and the contents sonicated with a Fisher model FB120 sonicator at 40% amplitude for 30 s. The tubes were centrifuged at 3,000 *g* for 5 min, and 200-µL aliquots of the supernatant was transferred into scintillation vials. After adding 5 mL of Bio-Safe scintillation cocktail and vortexing, tritium radioactivity of the samples was measured using a Beckman LS6500 liquid scintillation counter.

#### 2.2.5 Intracellular dopamine uptake in B35 neuroblastoma cells

B35 cells seeded in 48-well plates at a density of 3 × 10^4^ were allowed to grow to 80% confluence and then induced to differentiate through serum deprivation and addition of 0.5 mM dibutyryl cyclic-AMP. Prior to the uptake assay, the media was replaced with 200 µL of complete Neurobasal medium and cells incubated at 37°C for 3 h. Subsequent treatments were prepared at 10X final concentrations in Neurobasal media and the final reaction volume was adjusted to 250 µL.

To visualize cellular uptake of fluorescently labeled dopamine, B35 cells were incubated with dansyl-dopamine (FIVEphoton Biochemicals) at concentrations of 30–60 µM for 15–30 min. Following multiple washes with cold PBS, cellular dopamine fluorescence was observed at 315 nm excitation/515 nm emission in a Zeiss LSM 710 confocal microscope.

To quantitatively measure cellular uptake of dopamine, the specific activity of stock [^3^H]dopamine (20 Ci/mmol, 250 µCi stock) was diluted into 2 mL of cold 10 mM dopamine solution. Use concentrations ranging from 1–3,000 µM of the resulting low-specific activity dopamine in Neurobasal media were incubated with differentiated B35 cells for 30 min. Cells were quickly harvested into a Brandel Cell Harvester and washed five times with 2-mL changes of ice-cold PBS. Cells deposited onto glass-fiber filter paper were transferred into polypropylene tubes, 500 µL DDW was added followed by sonication for 15 s. Following centrifugation to pellet any debris, a 250 µL aliquot of the supernatant was transferred into scintillation vials, 5 mL Biosafe scintillation cocktail was added and radioactivity measured. Effectiveness of the washing steps was assessed by verifying the absence of significant radioactivity in blank samples that contained radioactivity but no cells, and by sampling of terminal wash effluents from sentinel samples.

#### 2.2.6 Intracellular dopamine microinjection and calcium imaging in U2-OS cells

We used U2-OS cells seeing their Soma is suitably large for microinjection and the cells do not natively express D_5_ dopamine receptors. The method was as previously reported with minor modifications (Brailoiu et al., 2012; Deliu et al., 2012). U2-OS cells were transfected with GFP-tagged human D_5_ receptor (DRD5) cDNA (OriGene.com, #RG202502) or pCMV6-AC-mGFP vector (OriGene.com #PS100040) using Turbofectin-8 transfection reagent (Origene.com #TF81001) according to manufacturer recommendations. Cells were used 24–48 h after transfection.

Intracellular microinjections were performed on U2-OS cells using Femtotips II, InjectMan NI2, and FemtoJet systems (Eppendorf.com) as reported previously (Brailoiu et al., 2011; Brailoiu et al., 2012). Pipettes were back-filled with an intracellular solution composed of 110 mM KCl, 10 mM NaCl, and 20 mM HEPES (pH 7.2) or solutions of the specific compounds to be tested. The injection time was 0.4 s at 60 hPa with a compensation pressure of 20 hPa to maintain the microinjected volume to <1% of cell volume (measured by microinjection of a fluorescent compound, Fura-2 free acid). The intracellular concentration of chemicals was determined based on the concentration in the pipette and the volume of injection. The cellular volume was estimated at 1,000 μm^3^ as previously described (Rubin et al., 1989).

Measurements of intracellular calcium concentration, [iCa^2+^], were performed as previously described (Brailoiu et al., 2011; Deliu et al., 2012). Cells were incubated in the dark with 5 μM Fura-2 a.m. (Invitrogen/ThermoFisher #F1221) in HBSS at room temperature for 45 min, washed three times with dye-free HBSS, and then incubated for 45 min to allow for complete de-esterification of the dye. Coverslips (25-mm diameter) were subsequently mounted in an open bath chamber (RP-40LP, Warner Instruments, Warneronline.com) on the stage of an inverted Nikon Eclipse TiE microscope equipped with a Perfect Focus System and a CoolSnap HQ2 CCD camera (Photometrics.com). During the experiments, the Perfect Focus System was activated. Fura-2 a.m. fluorescence (emission 510 nm), following alternate excitation at 340 and 380 nm, was acquired at a frequency of 0.25 Hz. Images were acquired and analyzed using NIS-Elements AR software (Ver. 3.1, Nikon). The ratio of the fluorescence signals acquired at 340/380 nm was converted to intracellular Ca^2+^ concentrations as described (Deliu et al., 2012).

#### 2.2.7 Detection of select transcript expression in clonal cell lines

Native striatal and cortical tissues are known to express the dopamine receptors and organic cation transporters implicated in this study. To gain insight into the relevant molecular participants in the effects observed in the clonal cells that we used, we used RT-PCR assays to analyze B35 and U2-OS cells for expression of appropriate dopamine receptors and the dopamine-preferring neuronal Uptake2-like transporter, PMAT (Engel et al., 2004; Duan and Wang, 2010). Total RNA from cells was extracted using RNeasy Mini Kit (Qiagen, #74106) according to the manufacturer’s instructions. DNase treatment was performed on RNA samples using RNase-Free DNase kit (Qiagen, #79254). cDNA was synthesized from 1 µg total RNA using the qScript cDNA synthesis Kit (Quantabio, #95047–100). Standard RT-PCR was conducted using primers for specific target mRNA, and up to 36 cycles of amplification were performed with the following reaction parameters: denaturation at 94°C for 30 s, annealing at 55°C for 30 s, and extension at 72°C for 1 min, plus an initial denaturation step of 5 min at 95°C and a final extension of 5 min at 72°C. The primer sets used in the experiments were as follows. For rat (B35 cells): *PMAT*: TTGGGTCCCTTGCTCTTT (forward), GGG​CAG​TAA​TCC​TGT​GTA​G (reverse); *DRD1*: GGG​AAT​TCA​GCT​AAG​CTG​GCA​CAA​GGC​AA (forward), GGC​TGC​AGA​ATG​GCT​GGG​TCT​CC (reverse); *DRD2*: GGG​AAT​TCG​CAG​CAG​TCG​AGC​TTT​CAG​A (forward), GGC​TGC​AGC​TCA​TCG​TCT​TAA​GGG​AGG​T (reverse); *DRD5*: AGT​CGT​GGA​GCC​TAT​GAA​CCT​GAC (forward), GCG​TCG​TTG​GAG​AGA​TTT​GAG​ACA (reverse). For human (U2-OS cells): *PMAT*: CTG​CTG​CCA​TAC​AAC​AGC​TTC​A (forward), CTC​CAC​CAG​GAC​GTT​GTT​CAG (reverse), *DRD1*: AAA​CCC​ACA​AGC​CCC​TCT​GA (forward), GAT​GAA​TTA​GCC​CAC​CCA​AAC (reverse); *DRD2*: GCG​GAC​AGA​CCC​CAC​TAC​AA (forward), AAG​GGC​ACG​TAG​AAG​GAG​A (reverse); *DRD5*: AAC​CTG​TGC​GTC​ATC​AGC​G (forward), CAG​ATC​CAT​GAG​GGG​GTT​T (reverse). The PCR products were examined using 2 µg of cDNA on a 2% agarose gel. Equal amounts of cDNA were used across test samples; however the intent of the experiment was to indicate presence *versus* absence, rather than a quantitative comparison, among the transcripts or tissues.

#### 2.2.8 Data analysis

In this study, with the exception of the calcium imaging data where each sample represents individual cells, sample replicates represent aliquots of cells or brain slices analyzed as a unit to yield one datum without regard to the number of individual animals from which the brain slices or primary cells were extricated. Cell lines necessarily derive from a single original source, thus providing comparable analyte baselines prior to experimental treatment. Each experiment was conducted with 2-4 replicate samples (cell or tissue slice aliquots) per data point and on multiple occasions to accumulate adequate sample sizes for statistical analysis (generally, N ≥ 6). Unless otherwise indicated, data are generally expressed as means with standard deviations (SD) or 95% confidence intervals (CI) for indicated sample sizes. Statistical analyses were performed with SPSS software (ibm.com/spss) or Prism 8.4.3 (GraphPad.com) using appropriate statistical procedures as described in the legend for each dataset. GraphPad Prism was used for curve-fitting and for graphical presentation of data. Each figure legend provides information on the type of analysis performed on the data and indications of statistical variance and significance as applicable. Statistical tests were two-sided, and statistical significance of mean differences was inferred at *p* < 0.05 or better.

## 3 Results

Given previous observations that dopamine and several D_1_-like receptor agonists can stimulate CDP-diacylglycerol formation in acute brain slice preparations (Undie, 1999; Sahu et al., 2009), that the D_1_-like receptor involved in the CDP-diacylglycerol response was mostly of the D_5_ sybtype (Sahu et al., 2009), that the preponderance of D_5_ receptor expression in forebrain tissues was intracellular (Voulalas et al., 2011), and knowing that CDP-diacylglycerol biosynthesis occurs intracellularly in the mitochondria or endoplasmic reticulum (Inglis-Broadgate et al., 2005), the questions that this study sought to address were: (i) are DAT-expressing presynaptic dopamine terminals necessary in order for dopamine to stimulate CDP-diacylglycerol biosynthesis in brain or neural tissues? (ii) if dopamine is acting at postsynaptic-type dopaminoceptive cells, might the postsynaptically expressed Uptake_2_ transporter and the intracellular cytoskeleton be involved in delivering extracellular dopamine to its intracellular sites of CDP-diacylglycerol regulation? (iii) can intracellular dopamine mediate a physiologically relevant D_5_ receptor-dependent response? The following results address these questions using experimental systems and ligands that are appropriate for each question.

### 3.1 Dopamine agonist effects on CDP-diacylglycerol in postsynaptic-type dopaminoceptive brain tissues and cells

**FIGURE 1:**
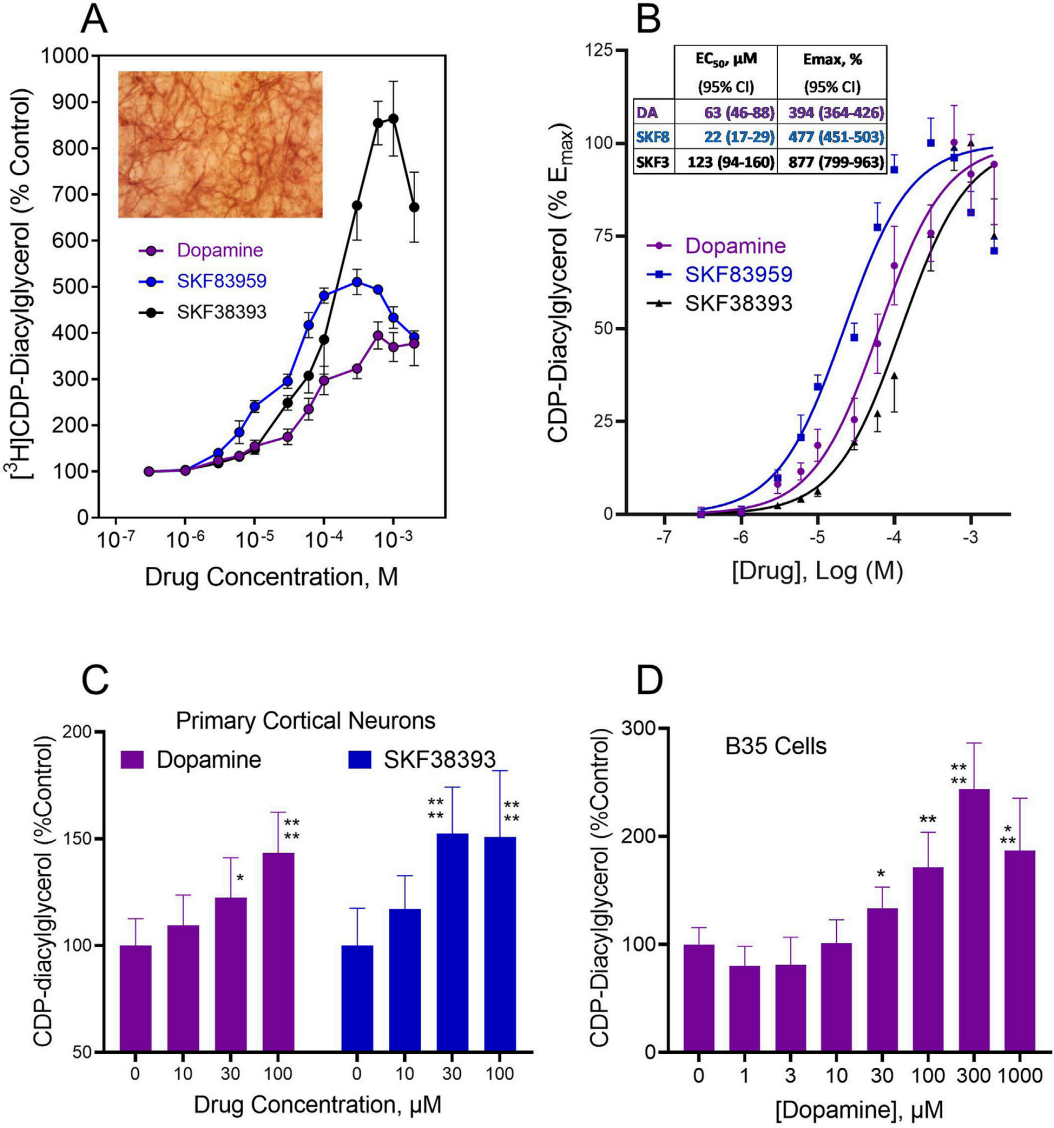
Dopamine agonist effects on CDP-diacylglycerol accumulation in cultured brain slices and neural cells that lack presynaptic dopamine terminals or endogenous dopamine. **(A)**. CDP-diacylglycerol accumulation in [^3^H]cytidine-prelabeled organotypic striatal slices following 90 min incubations with various concentrations of dopamine (N = 8) or the D_1_-like receptor agonists SKF38393 (N = 8) and SKF83959 (N = 4). [^3^H]CDP-diacylglycerol responses were converted to percentages relative to Control (608 ± 121 dpm/mg protein, N = 20) to yield the mean ± SD values shown. Each drug concentration-dependently increased [^3^H]CDP-diacylglycerol in the cultured slice preparations as compared by Two-Way ANOVA, *p* < 0.001 compared across drug concentrations; *p* < 0.01 compared among drugs. Insert micrograph illustrates the ordinary appearance of a MAP2-stained 7 day-cultured organotypic striatal slice that has thinned out to reveal networks of neurons within the organotypic matrix. **(B)**. Plot of data as net increase above control percent maximal response for each agent, thus the control response equals 0% and the maximal response equals 100%. A sigmoidal concentration-response function was fitted to the data to yield the fit curves shown. From this, pharmacological indices of potency (EC_50_) and efficacy (E_max_) were determined for each drug using GraphPad Prism. *Insert* shows the computed geometric mean EC_50_ and E_max_ values with 95% confidence intervals (95% CI) summarized for the test drugs. **(C)**. Agonist effects on [^3^H]CDP-diacylglycerol accumulation in frontal cortical neurons incubated with [^3^H]cytidine for 60 min. Each bar is mean ± SD for dopamine (N = 9) or SKF38393 (N = 6) as shown. Two-Way ANOVA analysis indicated differences between the effects of dopamine and SKF38393 (*p* < 0.05) as well as significant concentration-dependent effects of the drugs (*p* < 0.0001); subsequent posthoc tests for each drug showed significantly different mean effects at the 30–100 µM concentrations of dopamine or SKF38393. *p < 0.05; ****p < 0.0001 compared to respective control (0 µM) responses by Dunnett tests. **(D)** Effects of dopamine on CDP-diacylglycerol accumulation in B35 neuroblastoma cells incubated with [^3^H]cytidine and the indicated dopamine concentrations for 60 min. Each bar is the mean ± SD (N = 6). Drug concentrations of 30 µM and higher produced statistically significant increases in nucleolipid formation based on One-Way ANOVA analysis (*p* < 0.0001). *p < 0.05, **p < 0.01, ***p < 0.001, ****p < 0.0001 compared to the baseline (0 µM dopamine) group by *posthoc* Dunnett tests.

In the presence of presynaptic dopamine terminals, exogenous dopamine could be taken up by Uptake_1_/DAT into the cell and might then interact with the CDP-diacylglycerol biosynthetic apparatus to modulate synthesis of the nucleolipid. To test the hypothesis that the nucleolipid response occurs in cells that are postsynaptic with respect to dopamine terminals, we examined agonist efficacy in dopaminoceptive tissues and cell preparations that are reasonably devoid of presynaptic terminals and endogenous dopamine. These preparations included organotypic striatal tissues cultured *in vitro* for 7–10 days, primary cultures of cortical neurons maintained *in vitro* for 7–10 days, and B35 neocortical neuroblastoma cells demonstrated to express the D_5_ dopamine receptor. Preliminary dopamine radioimmunoassays showed that endogenous dopamine was undetectable in striatal slices after 24 h or longer in organotypic culture. Hence, experiments conducted on brain slices after 7–10 days in culture were considered to be devoid of presynaptic dopamine terminals and endogenous dopamine. As shown in Figure 1, dopamine significantly and concentration-dependently stimulated CDP-diacylglycerol accumulation in organotypic striatal slices, and these effects were mimicked by the D_1_-like receptor agonists SKF38393 and SKF83959 (Figure 1A). Each drug attained peak effects, after which higher drug concentrations produced decreasing responses–a pattern that is often observed for diverse dopaminergic responses and for dopamine-linked phospholipid responses in acute brain tissue preparations (Undie and Friedman, 1990). The CDP-diacylglycerol data were well-fitted to a sigmoidal concentration-response curve typical of receptor-dependent pharmacological responses (Figure 1B). Geometric means of pharmacological indices of potency (EC_50_) and efficacy (E_max_) estimated from these data are shown in the insert to Figure 1B. Summarizing, SKF83959 (EC_50_ = 22 µM) was apparently more potent than dopamine (EC_50_ = 63 µM) or SKF38393 (EC_50_ = 123 µM), whereas SKF38393 (E_max_ = 877%) was more efficacious than SKF83959 (E_max_ = 477%) or dopamine (E_max_ = 394%). Fold responses in this organotypic system were substantially greater than responses previously observed in acute brain slices, possibly because of the smaller basal CDP-diacylglycerol accumulation in the organotypic system, as well as the possibility that dopamine receptors in the cultured tissues may have undergone supersensitization as a result of endogenous dopamine deprivation.

Dopamine effects on CDP-diacylglycerol formation in cultured primary cortical neurons were examined in the absence of lithium chloride since the inositol phosphatase inhibitor was shown in preliminary assays to lack effect on enhancing CDP-diacylglycerol accumulation in tissue cultures (tissue culture media typically contain myo-inositol, thus possibly bypassing any lithium effect). As shown in Figure 1C, dopamine and SKF38393 concentration-dependently enhanced [^3^H]CDP-diacylglycerol accumulation in primary cortical neurons (Two-Way ANOVA, TWA, F_(3,52)_ = 20.17, *p* < 0.0001 for concentration-dependence, and F_(1,52)_ = 5.05, *p* < 0.05 for between-drug comparisons). Subsequent *posthoc* Dunnett analyses for dopamine showed significant effects at 30 µM (*p* < 0.05) and 100 µM (*p* < 0.0001) concentrations, while for SKF38393 statistical significance was detected at 30 µM (*p* < 0.0001) and 100 µM (*p* < 0.0001) concentrations compared to control.

The above native brain tissue effects were confirmed through testing on B35 rat cortical neuroblastoma cells which also express D_5_ dopamine receptors. Dopamine concentration-dependently induced CDP-diacylglycerol accumulation (One-Way ANOVA, OWA, F_(7,40)_ = 25.70, *p* < 0.0001) (Figure 1D). Drug concentrations of 30 µM and higher induced statistically significant [^3^H]CDP-diacylglycerol accumulation with net effects ranging from 35%–104% increases above baseline. Hence, presynaptic dopaminergic terminals or endogenous dopamine may not be required for exogenous dopamine agonists to stimulate CDP-diacylglycerol formation.

### 3.2 Effects of disrupting the microtubule transport system on agonist induction of CDP-diacylglycerol biosynthesis *versus* phosphoinositide hydrolysis in brain slices

**FIGURE 2:**
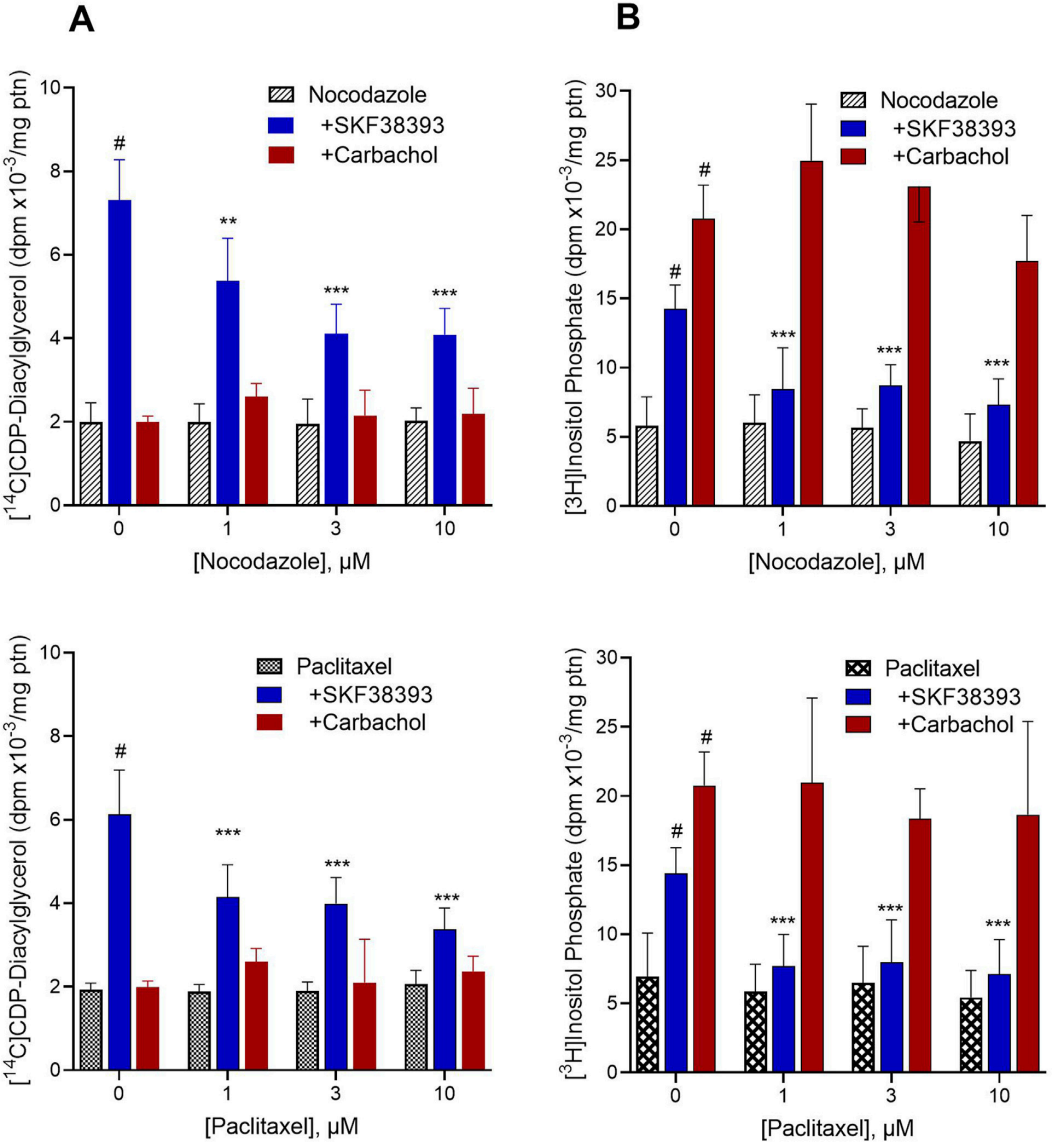
Effects of microtubule-disrupting agents (MDA) on drug-induced [^14^C]CDP-diacylglycerol and [^3^H]inositol phosphate accumulations. Freshly prepared rat striatal slices were concurrently incubated with [^14^C]cytidine and [^3^H]inositol to respectively label newly formed CDP-diacylglycerol and inositol phosphates. The results show the baseline effects of various concentrations of MDA alone, and the responses to 300 µM SKF38393 (N = 9) or 300 µM carbachol (N = 6) in the absence (0 µM) or presence of the MDA, nocodazole (Top, N = 9) and paclitaxel (Bottom, N = 9). Each bar is the mean ± SD of the responses expressed relative to protein (ptn) content of the brain slice aliquots (range 200–250 µg ptn). Data as shown for each graph were analyzed by Two-Way ANOVA of drug by MDA concentration followed by Dunnett tests to compare each agonist to MDA alone or each MDA concentration to its respective control (0 µM MDA). **(A)**. Left panel [^14^C]CDP-diacylglycerol effects. SKF38393 significantly induced CDP-diacylglycerol accumulation compared to nocodazole alone (*p* < 0.0001) or paclitaxel alone (*p* < 0.0001). Carbachol did not produce a statistically significant effect on CDP-diacylglycerol. Comparison of responses at each MDA concentration to the 0 µM MDA concentration (SKF38393 alone) showed significant inhibition of the SKF38393 response at 1–10 µM nocodazole or paclitaxel. **p < 0.01, ***p < 0.001 compared to the SKF38393 response in the absence of respective MDA. **(B)**. [^3^H]Inositol phosphate accumulation concurrently measured with the [^14^C]CDP-diacylglycerol assay as in panel **(A)**. SKF38393 and carbachol each significantly increased inositol phosphate accumulation compared to baseline (#*p* < 0.0001 compared to MDA alone). Comparison of responses at each MDA concentration to the SKF38393 response at 0 µM MDA by Dunnett test showed significant inhibition of the SKF38393 response by nocodazole and by paclitaxel. ***p < 0.001 compared to the SKF38393 response in the absence of respective MDA. Carbachol effects on inositol phosphate accumulation were not significantly altered by nocodazole or paclitaxel.

We explored whether dopamine, or some substance produced through the extracellular action of dopamine, may be transported through the microtubule system and thereby gain access to the microsomal compartment to stimulate CDP-diacylglycerol production. In this regard, it should be noted that dopaminergic stimulation of CDP-diacylglycerol precedes and probably accounts for dopaminergic stimulation of membrane-level inositol phosphate accumulation, whereas acetylcholine stimulates membrane inositol phosphate accumulation without needing to first promote CDP-diacylglycerol synthesis (Undie and Friedman, 1990; Tyeryar and Undie, 2007; Sahu et al., 2009). As such, if microtubule disruption inhibits dopaminergic effects on CDP-diacylglycerol and inositol phosphate without altering muscarinic receptor stimulation of inositol phosphate, then that would support a role for intracellular transport of dopamine agonist in order to induce CDP-diacylglycerol. To explore this, rat striatal slices were dual-labeled for 45 min with [^14^C]cytidine and [^3^H]inositol so as to simultaneously monitor CDP-diacylglycerol biosynthesis and PI metabolism, respectively. Prelabeled slices were incubated with microtubule-disrupting agents (MDA) for 10 min prior to addition of dopaminergic or cholinergic agonists for 60 min. As shown in the results (Figure 2), the microtubule disrupting agents nocodazole and paclitaxel did not significantly alter baseline accumulation of [^14^C]CDP-diacylglycerol or [^3^H]inositol phosphate. Exploring the standard responses of SKF38393 and carbachol compared to control in the absence of MDA treatment, SKF38393 (300 µM) significantly increased [^14^C]CDP-diacylglycerol (OWA with Dunnett, *p* < 0.0001 compared to baseline) whereas carbachol (300 µM) did not significantly alter CDP-diacylglycerol accumulation (Figure 2A). Examined by TWA (drug x MDA concentration), the CDP-diacylglycerol effects of SKF38393 were significantly blocked by nocodazole (*p* < 0.0001) and by paclitaxel (*p* < 0.0001). With regard to PI metabolism (Figure 2B), both SKF38393 and carbachol significantly increased inositol phosphate accumulation (OWA with Dunnett test, *p* < 0.0001 for each drug compared to respective MDA alone). The effects of SKF38393 were significantly reduced by nocodazole and by paclitaxel (TWA, *p* < 0.0001 for each MDA); however, carbachol-induced inositol phosphate responses were resistant to inhibition by microtubule disruption. Thus, dopaminergic stimulation of CDP-diacylglycerol and IP accumulations require an intact cellular microtubule transport system in contrast with cholinergic effects.

### 3.3 Effects of blocking Uptake_2_ on dopamine agonist-induced [^3^H]CDP-diacylglycerol biosynthesis

**FIGURE 3:**
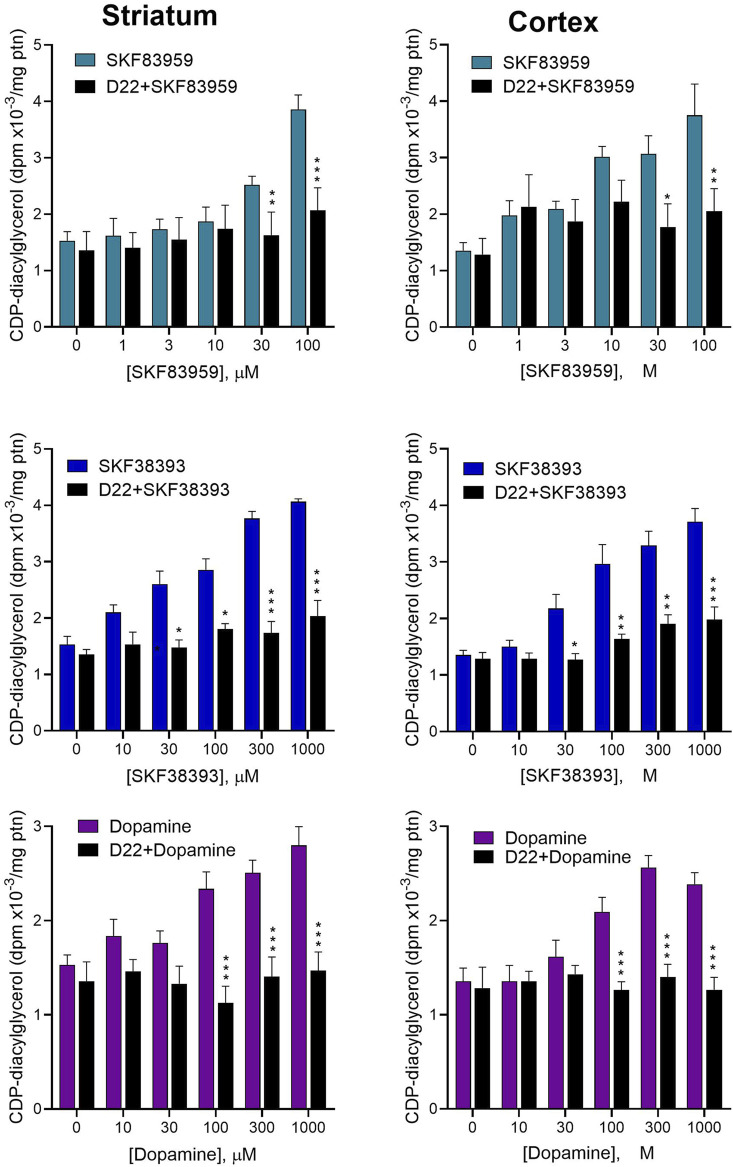
Effects of blocking Uptake_2_ with decynium-22 (D22) on dopamine agonist-induced CDP-diacylglycerol formation in acute brain slices. Freshly prepared brain slices from the rat striatum (Left panel) or frontal cortex (Right panel) were incubated with or without 30 µM D22 and tested for CDP-diacylglycerol accumulation in response to indicated concentrations of SKF83959 (N = 6), SKF38393 (N = 6) or dopamine (N = 9). The 30 µM concentration was selected for use in order to ensure adequate penetration into the 350 µm-thick slices. Data from multiple experimental runs were normalized against control (no drug) values and pooled for statistical analysis and graphical presentation of each treatment pair (drug *versus* D22+drug) for each brain region. Each bar is the mean ± sem of CDP-diacylglycerol expressed relative to average protein (ptn) content of the slices for the indicated sample sizes. Based on the outcomes of the six respective Two-Way ANOVA tests, D22 significantly reduced the responses to dopamine (*p* < 0.0001), SKF38393 (*p* < 0.0001) and SKF83959 (*p* < 0.0001) on CDP-diacylglycerol accumulation in both the striatum and the cortex; moreover, the main effects of each drug were significantly concentration-dependent (*p* < 0.001 for each drug in each tissue). Subsequent Bonferroni paired comparisons performed at each drug concentration are shown: *p < 0.05, **p < 0.01, ***p < 0.001 comparing drug + D22 to drug alone.

We next tested whether functionally relevant dopamine internalization occurs in native brain tissue preparations to affect CDP-diacylglycerol biosynthesis. For this, we assayed agonist-induced CDP-diacylglycerol accumulation in fresh brain slice preparations incubated with the postsynaptic Uptake_2_ inhibitor, decynium-22 (D22). As seen in Figure 3 D22 at 30 µM concentration inhibited the effects of dopamine, and of the D_1_-like receptor agonists SKF38393 and SKF83959, on [^3^H]CDP-diacylglycerol accumulation in striatal and cortical brain tissues (TWA, *p* < 0.01 for each agonist effect in the presence of D22 compared to the respective agonist effect alone). These observations suggest that Uptake_2_-mediated ligand uptake may be involved in the steps to dopaminergic modulation of neural [^3^H]CDP-diacylglycerol biosynthesis.

### 3.4 Intracellular dopamine uptake by neural cells and effects of Uptake_2_ blockade

The foregoing CDP-diacylglycerol experiments suggest that postsynaptic plasmamembrane dopamine uptake into neural cells could be critical for subsequent stimulation of CDP-diacylglycerol biosynthesis. We directly addressed this idea in two sets of experiments that measured dopamine uptake - the first set was based on primary cortical cells in culture, and the second set used B35 neuroblastoma cells expanded from the same stock to allow for quantitative comparisons across experiments.

**FIGURE 4:**
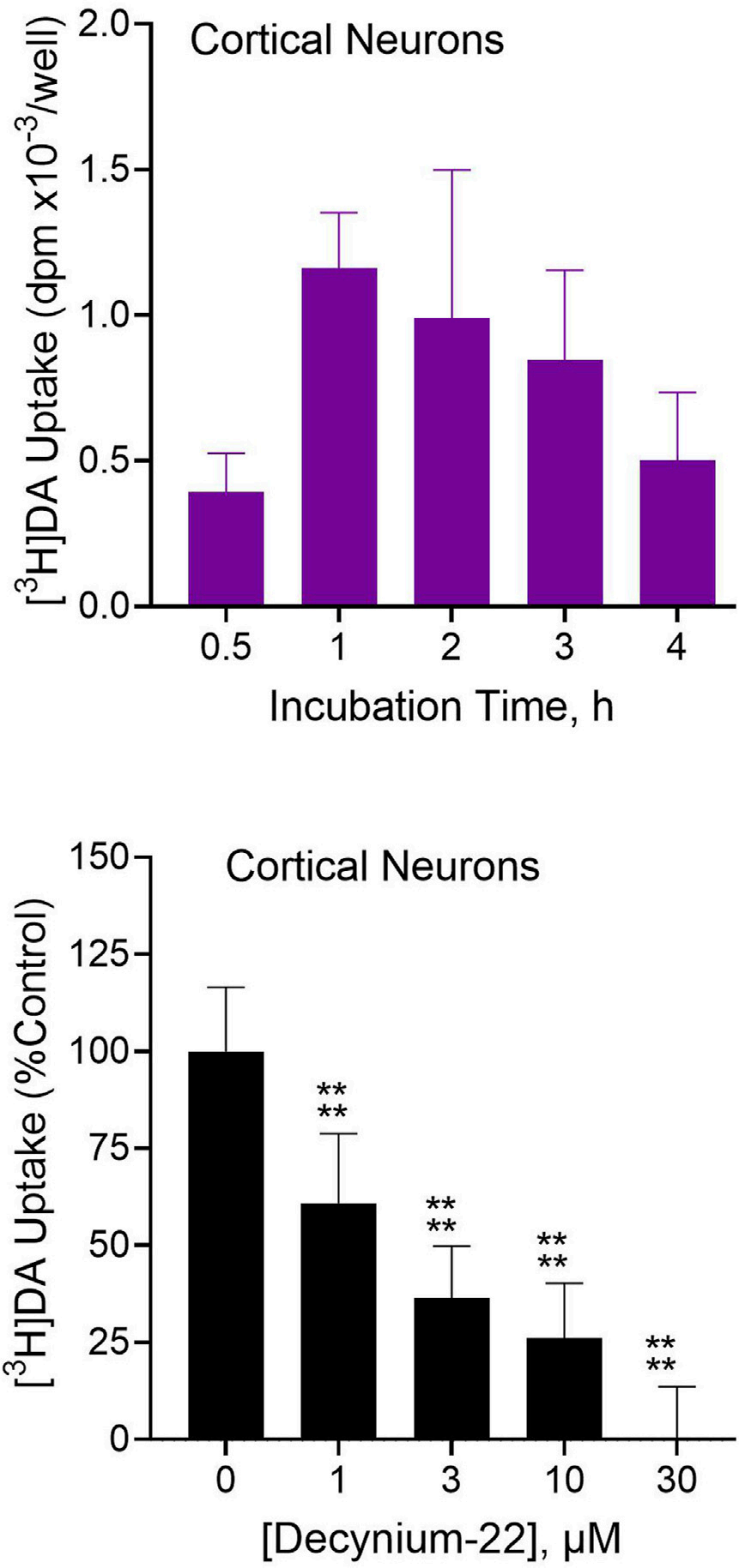
[^3^H]Dopamine ([^3^H]DA) uptake into rat primary cortical neurons and effects of Uptake_2_ inhibition. Dissociated rat cortical neurons were cultured *in vitro* for 7 days and then used for testing. *Top*: Cultured cortical neurons were incubated with 100 µM [^3^H]dopamine for indicated times followed by analysis of intracellular tritium uptake. Each bar is the mean ± SD (N = 3). Substantial and time-dependent [^3^H]DA uptake was observed in the cultured primary cortical neurons. *Bottom*: Primary cortical neurons were incubated with 100 µM [^3^H]DA in the presence of indicated concentrations of D22 followed by analysis of [^3^H]DA uptake. Nonspecific uptake was defined with 30 µM D22. Data from multiple experimental runs was normalized by converting to percentages relative to uptake in the absence of D22 (equated to 100%). Each bar is the mean ± SD (N = 12). Data were analyzed by One-Way ANOVA followed by Dunnett test. ****p < 0.0001 compared to uptake in the absence of D22.

In 7-day-cultured rat primary cortical neurons incubated with 100 µM [^3^H]dopamine, the tritiated dopamine was taken up into the cells in a time-dependent manner, with apparent peak uptake occurring within 1 hour of exposure (Figure 4, top). As further shown (Figure 4, bottom), neuronal uptake of [^3^H]dopamine was significantly inhibited by 1–10 µM concentrations of the Uptake_2_ inhibitor, D22 (OWA, *p* < 0.0001). Thus, native brain neurons can take up extracellular dopamine in an Uptake_2_-dependent manner.

**FIGURE 5:**
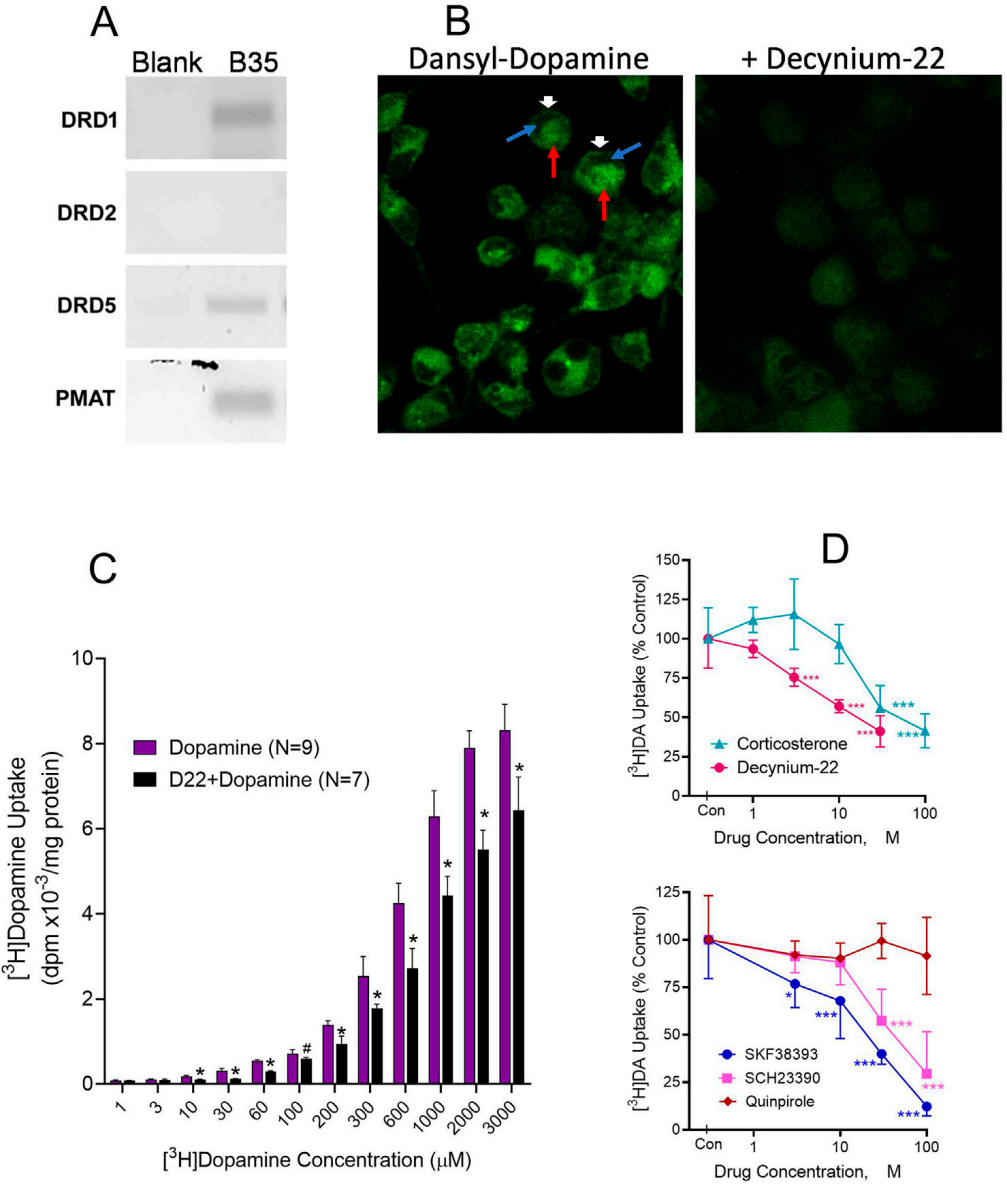
Intracellular dopamine (DA) uptake and effects of competing ligands and Uptake_2_ modulation. **(A)**. Rat B35 cortical neuroblastoma cells demonstrably expressing PMAT (in addition to D_1_ and D_5_ receptors but not D_2_ receptor) were used. **(B)**. Fluorescent dansyl-labeled DA uptake in B35 cells incubated for 30 min with 30 µM dansyl-dopamine only (Left) or 30 µM dansyl-dopamine in the presence of 10 µM decynium-22 (Right). Cells were visualized under confocal microscopy. Intracellular green fluorescence indicates dansyl-dopamine entry and retention in the B35 cells. Note the discrete intracellular distribution of the dopamine fluorescence in incubations with dansyl-dopamine, especially in the absence of decynium-22. Arrows show: Dansyldopamine fluorescence in cell membrane (White arrowhead); Cellular subregion of low dansyldopamine fluorescence (Blue arrow); Cellular subregion of high dansyldopamine fluorescence (Red arrow). **(C)**. Cellular [^3^H]DA uptake in B35 cells incubated with indicated molar concentrations of total (cold + radiolabeled) dopamine in the presence or absence of 10 µM decynium-22 (D22). Nonspecific uptake was defined with 30 µM D22. Each bar is the mean ± SD (N = 7–9). Two-Way ANOVA showed significant effects of dopamine concentration (*p* < 0.0001) and of D22 pretreatment (*p* < 0.0001) on [^3^H]dopamine uptake. *p < 0.001 compared to the paired concentration of dopamine alone. **(D)**. Effects on [^3^H]DA uptake of Uptake_2_ inhibitors D22 and corticosterone (Top) and competition by DA ligands (Bottom). Indicated concentrations of each uptake inhibitor compound were tested against 100 µM [^3^H]DA in B35 cells. Nonspecific uptake was defined with 300 µM SKF38393. Each point is the mean ± sem (N = 6). Two-Way ANOVA was performed for the competing agents followed by Dunnett testing to compare the concentration effect of each agent to its control ([^3^H]DA alone). *p < 0.05, ***p < 0.001 compared to Control (Con [^3^H]DA alone) for each drug treatment by Dunnett test.

For more reproducible analyses across experiments, we turned to B35 cortical neuroblastoma cells. In addition to expressing D_5_ receptors, B35 cells express the Uptake_2_ molecular component, PMAT (Figure 5A). First, we used fluorescently tagged dopamine, dansyl-dopamine, to image dopamine uptake. Incubations of B35 cells with dansyl-dopamine 30 µM for 30 min resulted in substantial uptake of the compound into the cells as shown by the intracellular distribution of dansyl fluorescence (Figure 5B, Left image). Cellular dansyl-dopamine fluorescence was nonuniform across the cell, which may suggest differential distribution of the compound among cellular subcompartments or organelles. In the presence of 10 µM D22, dansyl-dopamine uptake was substantially reduced as demonstrated by the loss of fluorescence intensity across D22-treated cells (Figure 5B, Right image).

Using [^3^H]dopamine as substrate for the uptake assay, we conducted quantitative saturation-mode assays (30-min incubations). Tritiated dopamine was taken up into B35 cells in a concentration-dependent (*p* < 0.0001) and apparently saturable manner. Robust uptake was observed at 30 µM and higher concentrations of dopamine, with peak effects occurring by 1–2 mM concentrations (Figure 5C). On exploring the effects of the Uptake_2_ inhibitor D22 on [^3^H]dopamine uptake, a fixed concentration of 10 µM D22 tested against various concentrations of dopamine resulted in significant inhibition of dopamine uptake with a rightward shift in the dopamine concentration-response curve (Figure 5C). Uptake inhibition by D22 was statistically significant at dopamine concentrations of 10 µM and higher. Between 30 μM and 1,000 µM dopamine [^3^H]dopamine uptake increased 2-3-fold for each 0.5 log increase in dopamine concentration. An attempt to fit a Hill function to the data resulted in substantially large but comparable Hill coefficients of 7.72 and 6.61 for the dopamine and D22+dopamine datasets, respectively. Note that nonspecific uptake in this experiment was defined by 30 µM D22, and it is possible that other factors such as the presence of DMSO may have been at play to influence the total uptake (see competition experiments below and Supplementary Figure S1).

We examined select compounds for competitive inhibition of dopamine uptake–Uptake_2_ inhibitors D22 and corticosterone, and several dopaminergic agents known to exert stimulatory (SKF38393 and SKF83959), inhibitory (SCH23390) and nonsignificant (quinpirole) effects on CDP-diacylglycerol. Multiple concentrations of the various agents were tested against a fixed 100 µM concentration of [^3^H]dopamine. As shown in Figure 5D (top graph), D22 was more potent than corticosterone in inhibiting [^3^H]dopamine uptake (TWA, *p* < 0.0001 for drug; *p* < 0.0001 for drug concentration). Moreover, the D_1_-like receptor agonists SKF38393 and SKF83959, and antagonist SCH23390, significantly and concentration-dependently competed against dopamine uptake, whereas the D_2_-like receptor agonist quinpirole was without effect (Figure 5D, bottom). Nonspecific uptake in these competition experiments was defined with 300 µM SKF38393. The highest concentration of D22 tested in the experiments was limited to 30 µM because higher concentrations produced higher levels of apparent dopamine uptake (see Supplementary Figure 1A). This paradoxical D22 effect was later found to be associated with the DMSO solvent used for dissolving D22. We tested DMSO by itself and found substantial concentration-related effects to increase dopamine “uptake” by increasing the association of the dopamine label with the cell membrane (See Supplementary Figure 1B). Based on the observations, the highest concentration of DMSO that could be tolerated, therefore, was 0.3%. Given D22’s 10 mM solubility in DMSO, 30 µM D22 which contained 0.3% DMSO was the highest concentration of D22 that contained a low-enough concentration of DMSO to be used without much concern for the DMSO artifactually raising the association of the dopamine label with the cell membrane.

### 3.5 Effects of intracellular dopamine microinjection on D_5_ receptor-dependent calcium mobilization

**FIGURE 6:**
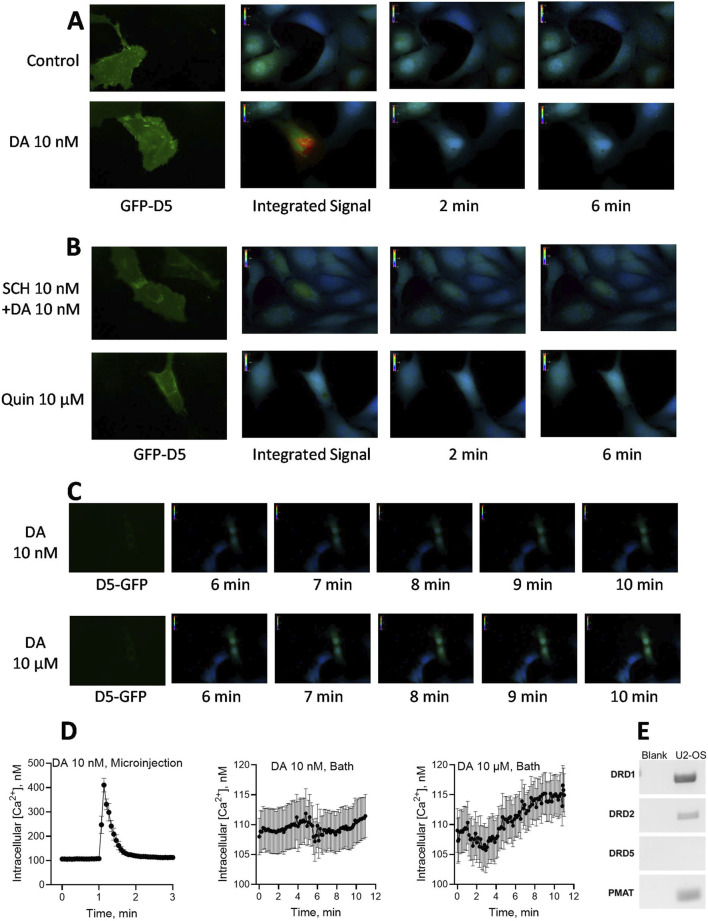
Effects of dopamine (DA) microinjection or bath application on intracellular free calcium concentrations [iCa^2+^] in hDRD5 receptor-expressing cells. **(A)**. Human U2-OS osteosarcoma cells natively expressing DRD1 and PMAT, but not DRD5, were pre-transfected with GFP-fluorescent human DRD5 receptor (green GFP-D5 fluorescence). Dopamine and other treatments were microinjected into the cell Soma followed by continuous 0.25 Hz recording of [iCa^2+^] signal as indicated by Fura-2AM. fluorescence. Pictograms of [iCa^2+^] from respective representative cells are shown (at least 12 transfected cells were tested with similar outcomes, and average results from 12 cells are shown in part D below). The control cell tested in parallel (top panel) was microinjected with buffered media alone, while the DA injected cell received 10 nM final intracellular concentration of DA. The [iCa^2+^] signal integrated over a 6 min observation window as well as point observations at 2 and 6 min, are shown. **(B)**. Effects of co-microinjection of SCH23390 (SCH) 10 nM with dopamine 10 nM (Top panel) or of quinpirole microinjection (up to 10 μM, bottom panel) on [iCa^2+^] response. SCH23390 blocked the iCa^2+^ response to dopamine, while the D_2_ agonist quinpirole (Quin) was without effect. **(C)**. Pictograms from representative experiments testing the effects of bath-applied DA on iCa^2+^ mobilization in U2-OS cells. Bath application of 10 nM dopamine had no effect (Top panel), while 10 µM dopamine monitored over 11 min revealed a slowly rising wave of [iCa^2+^] response that was highest toward the latest 11 min time point observed (Bottom panel). **(D)**. Quantified observations and temporal patterns of responses for microinjected *versus* bath applied dopamine. Continually recorded [iCa^2+^] data resampled at 0.133 min intervals from 12 experiments are shown for microinjected DA 10 nM (Left graph, peak effect = 416%) in contrast with the effects of bath-applied DA 10 nM (Middle graph) and bath-applied DA 10 µM (Right graph). For bath-applied DA 10 μM, the average of the three observations made in the last 12 s of recording (106.2%, N = 12) was compared to the average of the three observations made in the first 12 s (0.2 min) of recording as the baseline (100%, N = 12); the comparison indicated a statistically significant increase in bath-applied dopamine-stimulated [iCa^2+^] response (paired *t*-test, *p* = 0.019). The experimental system could not support extension of observation period past the 11th minute. **(E)** Expression indications for various dopamine receptors and PMAT in U2-OS cells. While D_1_ and D_2_ receptors as well as PMAT were expressed, there was no indication of D_5_ receptor expression in the U2-OS cells.

We next examined the hypothesis that intracellular dopamine can mediate a physiologically relevant response via intracellular D_5_ receptors. Given that the D_5_ receptor has been implicated in dopamine’s effects on cellular calcium mobilization (Lezcano and Bergson, 2002; Baufreton et al., 2003; So et al., 2009), and on CDP-diacylglycerol signaling (Sahu et al., 2009), we took advantage of this information in designing the experiments. We selected to use human U2-OS osteosarcoma cells because they have large enough Soma for microinjection; these cells natively express the D_1_ receptor but not the D_5_ dopamine receptor (Figure 6). A culture of human U2-OS cells was transfected with GFP-tagged human DRD5 receptor clone and individual suitably sized cells were confirmed for DRD5 expression (GFP fluorescence) just before microinjection and calcium imaging. As shown in the results (Figure 6), microinjection of dopamine 10 nM into D_5_-transfected cells elicited a fast and robust intracellular calcium signal that appeared soon after microinjection, peaked within 2–5 s, and then dissipated (Figures 6A,D Left graph). Microinjection of buffer alone into DRD5-transfected cells (Figure 6A, Control) produced no iCa^2+^ response. Nor was iCa^2^ elicited by microinjection of dopamine into sham-transfected (non-D_5_ receptor-expressing) U2-OS cells, thus indicating that the natively expressed D_1_ receptor in these cells was incapable of producing the iCa^2+^ response. Quantified, the dopamine response corresponded to a 316% increase in iCa^2+^ concentration above baseline levels (Figure 6D, Left graph). The iCa^2+^ response to microinjected dopamine was blocked by co-microinjection or 5-min pre-microinjection of 10 nM SCH23390 (Figure 6B). Microinjection of the D_2_-like receptor agonist, quinpirole, at concentrations of 10 nM–10 μM, did not produce a measurable change in iCa^2+^ concentrations (Figure 6B). Interestingly, bath application of 10 nM dopamine did not elicit any iCa^2+^ response (Figures 6C,D Middle graph), whereas a higher concentration of 10 µM dopamine elicited a slowly rising iCa^2+^ response that was noticeable by 1 min and increased gradually through 11 min–the longest observation period that the experimental system could allow (Figures 6C,D, Right graph). The level of iCa^2+^ mobilization by the 11th minute following bath application was statistically significant (Paired *t*-test, *p* < 0.05) compared to the baseline. The demonstrated expression of PMAT in the U2-OS cells (Figure 6E) is consistent with the effectiveness of bath-applied dopamine, seeing PMAT could intake the extracellular ligand to mediate intracellular iCa mobilization as found by others upon testing in dissociated neuronal preparations (Lezcano and Bergson, 2002; Baufreton et al., 2003).

## 4 Discussion

**FIGURE 7:**
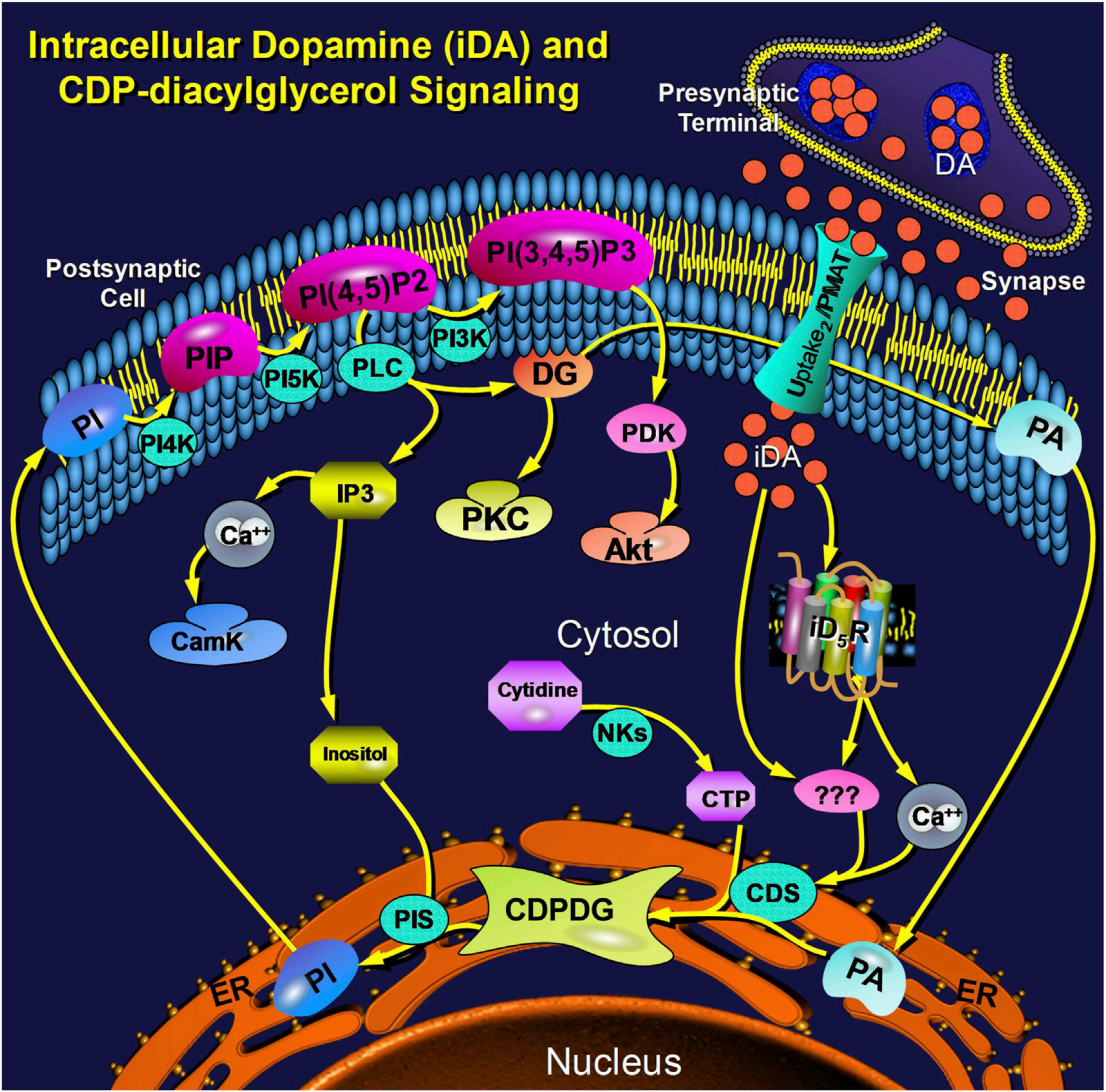
Schema illustrating an integrated view of cellular dopamine uptake and postsynaptic intracellular signaling via CDP-diacylglycerol. Dopamine (DA) is released from presynaptic nerve terminals into the synaptic cleft and extracellular space where threshold concentrations of the transmitter trigger its active transport via the postsynaptic Uptake_2_/PMAT transporter into the postsynaptic cell. Postsynaptic intracellular dopamine (iDA) interacts with intracellular D_5_-type receptors (iD_5_R), probably anchored to the bilaminar membrane of a subcellular organelle, to elicit a cytosolic calcium response. The elevated calcium, probably in concert with other presently unknown mediators/mechanisms (shown as triple question marks), may then activate CDP-diacylglycerol synthase (CDS) to increase output of CDP-diacylglycerol (CDPDG). While the nature of CDS activation is presently unclear, the steps involving the conversion of CDP-diacylglycerol to PI and the subsequent reactions of the membrane phosphatidylinositides are well established; these components are shown here in context to illustrate their relatedness to the rate-limiting step of CDP-diacylglycerol biosynthesis. Other Abbreviations: PA (phosphatidic acid), NKs (nucleotide kinases), PIS (phosphatidylinositol synthase), PI (phosphatidylinositol), PIP (PI phosphate), PI-4,5-P2 (PI-4,5-bisphosphate), PI-3,4,5-P3 (PI-3,4,5-trisphosphate), PI4K (PI-4-kinase), PI5K (PI-5-kinase), PI3K (PI-3-kinase), IP3 (inositol trisphosphate), CamK (calmodulin kinases), DG (diacylglycerol), PKC (protein kinase C), PDK (PI-3,4,5-P3-dependent kinase), PMAT (plasmamembrane monoamine transporter).

This study explored cellular mechanisms by which extracellular dopamine modulates intracellular CDP-diacylglycerol biosynthesis and subsequent phosphatidylinositol signaling. The biochemical significance of CDP-diacylglycerol as precursor to phosphatidylinositol biosynthesis is well characterized (Heacock and Agranoff, 1997; Lykidis et al., 1997; Monaco and Feldman, 1997; Shen and Dowhan, 1997). Nevertheless, there is yet no schema to explain the molecular mechanisms by which dopamine agonists modulate CDP-diacylglycerol formation at intracellular compartments. Nor has a physiologically relevant role been associated with the predominantly perikaryal and intracellular distribution of forebrain D_5_ dopamine receptors (Ciliax et al., 2000; Khan et al., 2000; Oda et al., 2010; Voulalas et al., 2011). Our study suggests a possible stratagem linking these entities and responses beginning with active transport of extracellular dopamine into the cell. The essential elements of this model are illustrated in Figure 7. While many details remain to be resolved in further studies, the present results open a new path to ongoing explorations of dopamine signaling and its contributions to brain function and dysfunction.

### 4.1 Dopamine-sensitive CDP-diacylglycerol biosynthesis is a postsynaptic receptor-mediated cellular response in dopaminoceptive tissues

Postsynaptic-type dopaminoceptive striatal slice cultures demonstrably devoid of presynaptic dopaminergic terminals or endogenous dopamine content yielded robust induction of [^3^H]CDP-diacylglycerol in response to added dopamine. Similarly, cortical primary neurons or neuroblastoma cells supported exogenous dopamine-induced CDP-diacylglycerol accumulation. In these various tissue or cell types, the dopamine effects were dose-dependent, mimicked by D_1_-like receptor agonists but not by quinpirole, and blocked by SCH23390 consistent with the D_1_-like/D_5_ receptor selectivity of the nucleolipid response (Undie, 1999; Sahu et al., 2009). Both dopamine D_5_ receptors and the *cds1*/*cds2* transcripts that catalyze CDP-diacylglycerol synthesis are known to be expressed in the studied neural tissues. Hence, these results provide evidence that the dopaminergic actions to promote CDP-diacylglycerol biosynthesis are occurring at postsynaptic-type dopaminoceptive cells rather than in cells that supply presynaptic dopamine terminals, thus ruling out direct DAT participation in the intracellular CDP-diacylglycerol response to dopaminergic agonists.

### 4.2 CDP-diacylglycerol formation requires intact cytoskeletal transport system

The microtubule cytoskeleton articulates with the cell membrane and various intracellular organelles, and is continuous with the ER which is the major site of phosphatidylinositol-associated CDP-diacylglycerol biosynthesis (Davies, 1993; Toomre et al., 1999; Nogales, 2000; Kevenaar and Hoogenraad, 2015). Microtubule-disrupting agents such as paclitaxel and nocodazole can provide insights on the dependence of a pharmacological response on mass transport through the cytoskeletal microtubule system (Dumontet and Sikic, 1999; Downing, 2000). Moreover, microtubule disruption can prevent the recruitment of intracellular receptors including D_1_-like receptors (Remy-Kristensen et al., 2000; Kruse et al., 2003). Actions and events localized to the cell membrane are less susceptible to abrogation by cytoskeletal disruptors compared to responses that occur only after cytoskeletal payloads have been delivered to their subcellular targets. The observation that paclitaxel or nocodazole prevented SKF38393-induced CDP-diacylglycerol and IP accumulation implicates subcellular translocation of dopamine agonist, or of a substance produced through the membrane action of the dopamine agonist, as important for dopaminergic modulation of CDP-diacylglycerol formation and thus the facilitation of PIP2 formation for enhanced IP accumulation. This contrasts with the actions of the cholinergic system where carbachol failed to enhance CDP-diacylglycerol and the microtubule disrupters failed to alter carbachol-induced IP accumulation, consistent with the membrane-level site of muscarinic receptor action. Consistent with previous suggestions that dopaminergic stimulation of inositol phosphates may depend on preceding dopaminergic facilitation of CDP-diacylglycerol biosynthesis (Undie, 1999; Sahu et al., 2009), the present results further imply that the microtubule network may receive and deliver a dopamine-engendered payload to an intracellular site in order for dopamine to facilitate CDP-diacylglycerol formation. An involvement of the microtubular transport network might also facilitate chaperoned delivery of payload to specific substructures of subcellular organelles.

### 4.3 Dopamine is internalized through a saturable transmembrane transport system

We next addressed the question whether a postsynaptic plasmamembrane transporter could intake dopamine into the cell, possibly guided into the microtubule network, for delivery to appropriate intracellular sites to participate in actions leading to enhanced CDP-diacylglycerol biosynthesis. Using fluorescent dansyl-dopamine which is recommended by the vendor for dopamine uptake studies, we observed strong intracellular dansyl-dopamine fluorescence in B35 cells with subcellular regions of high and low fluorescence intensity. Further, B35 cells avidly took up tritiated dopamine in a concentration-dependent and saturable manner. Thus, facilitated transport may provide a plausible mechanism for extracellular dopamine to gain access to the intracellular environment and possibly reach the sites of CDP-diacylglycerol biosynthesis. The apparent nonuniform distribution of dansyl-dopamine fluorescence intensity across the cell Soma may suggest differential accumulation of internalized dopamine among subcellular compartments. The identities of such cellular subcompartments that may receive internalized dopamine or harbor intracellular D_5_ receptors are currently unknown and are the subject of forthcoming studies.

Cellular [^3^H]dopamine uptake was strongly inhibited by decynium-22 which competitively inhibits the postsynaptic monoamine/organic cation transporters PMAT and OCT3 (Hill et al., 2011; Fraser-Spears et al., 2019). These transporters as a class, and PMAT in particular, are thought to be equivalent to the classical Uptake_2_ postsynaptic monoamine transporter (Duan and Wang, 2010; Wang, 2016; Vieira and Wang, 2021). Their transport function is membrane potential-sensitive and Na^+^-independent. Both transporters are inhibited by decynium-22, while OCT3 is more sensitive to corticosterone (Hayer-Zillgen et al., 2002; Engel et al., 2004; Matthaeus et al., 2015). PMAT is of further interest because of its strong expression in the brain, with additional expression in the small intestine, kidney, and heart (Engel et al., 2004; Itagaki et al., 2012). Decynium-22 was more potent than corticosterone in blocking [^3^H]dopamine uptake, implying a greater role for PMAT than OCT3 in dopamine transport, as previously reported (Duan and Wang, 2010; Wang, 2016; Vieira and Wang, 2021). The observation that the decynium solvent, DMSO, could promote nonspecific dopamine ligand association with the cell membrane should raise caution in use of DMSO-solubilized decynium, especially at concentrations higher than 30 µM (corresponding to DMSO concentrations of 0.3% or higher).

The results further show that dopamine uptake occurred within time frames and concentration ranges that are characteristic for induction of CDP-diacylglycerol (Undie, 1999; Sahu et al., 2009). The pattern suggests that the uptake mechanism is triggered by micromolar concentrations of extracellular dopamine with a threshold of about 10–30 µM. While the present saturation design of the uptake studies was chosen to correspond to the design of the CDP-diacylglycerol experiments, the observations are consistent with the reported 201–466 µM ranges for the Km of PMAT-mediated dopamine transport (Shirasaka et al., 2017). The physiopathologic relevance of this system may be highlighted by observations that high micromolar to millimolar extracellular levels of dopamine are achievable during high phasic stimulation, following drug-induced dopamine release, after drug-induced presynaptic re-uptake inhibition, or in certain hyperdopaminergic disorders such as schizophrenia and amphetamine psychosis (Di-Chiara and Imperato, 1988; Camp et al., 1994; Breier et al., 1997). Hence, a high-capacity transport system would be functionally critical during high rates of physiologic presynaptic stimulation or following pathologic exposure to release-enhancing or reuptake-blocking drugs.

### 4.4 Intracellular dopamine can mediate physiologically relevant cellular responses via intracellular D_5_ receptors

To query whether the presumed intracellular action of dopamine involves co-optation of a cognate intracellular receptor, we focused on the D_5_ receptor given its predominantly intracellular expression (Voulalas et al., 2011), its prior implication in CDP-diacylglycerol signaling (Sahu et al., 2009), and its role in neuronal calcium release in response to dopamine D_1_-like receptor agonists (Lezcano and Bergson, 2002; Baufreton et al., 2003; Ming et al., 2006). Note that while D_1_ receptor expression seemed robust, as indicated in the data (Figure 6E), the U2-OS cell line did not natively express D_5_ receptors as determined by RT-PCR analysis, hence we could assess the dependence of dopamine-mediated intracellular calcium response on the expression of transfected human D_5_ receptor. The immediate and dramatic release of cytosolic calcium in response to microinjection of nanomolar concentrations of dopamine into DRD5-transfected cells, and blockade of the response by SCH23390, implies that intracellular dopamine and intracellular D_5_ receptors are capable of interacting to elicit a physiological effect which, at the least, includes intracellular calcium mobilization. The specific organelle harboring such intracellular receptors is currently unknown. Other studies of intracellular G-protein-coupled receptors have found cognate signaling from endosomal, nuclear, microsomal, or other subcellular compartments (Brailoiu et al., 2014; Wu and O’Connell, 2015; Nash et al., 2019). We intend to explore the D_5_-receptor’s organellar anchorage and its access to dopamine in future studies.

It was noted that bath application of dopamine produced a slow but cumulative intracellular calcium response in D_5_-transfected cells but not in wildtype cells lacking D_5_ receptors. Given that U2-OS cells natively expressed PMAT, it is likely that the drug was transported by wild-type PMAT into the cell to interact with transfected D_5_ receptors. The slower rate of the iCa^2+^ response to bath-applied drug reflects previous timeline observations in primary cortical or hippocampal neurons (Lezcano and Bergson, 2002), and may be the result of a time-dependent transporter-mediated entry of dopamine into the cell. Indeed, as previously noted, dopaminergic-induced CDP-diacylglycerol and IP responses require up to 60 min to attain peak effects (Undie and Friedman, 1990; Undie, 1999), and in the present study at least 30 min was required to achieve steady-state dopamine uptake in B35 cells. Hence, contrasted with microinjected dopamine which instantaneously reaches intracellular sites to generate an immediate response, the slower, smaller, and prolonged effects bath-applied drug probably result from the relatively slow transmembrane transport of the drug which thus serves as a common time-limiting factor in these systems.

### 4.5 Efficacy of compounds to modulate CDP-diacylglycerol biosynthesis may depend on their intracellular uptake

A major implication of this study is the suggestion that compounds other than dopamine may also need to be taken up into the cell in order to modulate CDP-diacylglycerol biosynthesis. The D_1_-like dopaminergic agonists SKF38393 and SKF83959, and antagonist SCH23390, effectively competed against [^3^H]dopamine uptake, whereas the D_2_-like agonist quinpirole was without effect on D22-sensitive dopamine uptake. These observations are interpreted to indicate that compounds that effectively compete against dopamine are also taken up by the Uptake_2_/PMAT system that internalizes dopamine. These results correlate with the known efficacious effects of the D_1_-like compounds and the lack of effect of quinpirole on CDP-diacylglycerol or phosphatidylinositol signaling in brain tissue (Undie and Friedman, 1990; Undie, 1999; Jin et al., 2003). Thus, we surmise that drugs capable of modulating CDP-diacylglycerol signaling, whether agonistic or antagonistic in pharmacology, may have to be transported into the cell in order to access the relevant target and demonstrate activity. It would be interesting to elucidate how this pharmacological double-gating–suitability as Uptake_2_ substrate *and* intracellular D_5_ receptor efficacy–might shed light on hitherto enigmatic physio-pharmacologic phenomena such as “spatially selective signaling” or “functional selectivity” and other forms of biased agonism, at least with regard to D_1_-like receptor agents (Mailman, 2007; Ryman-Rasmussen et al., 2007; Irannejad et al., 2017; Radoux-Mergault et al., 2023).

### 4.6 Summary and conclusion

The results of this study provide evidence that dopamine is actively transported into postsynaptic-type dopaminoceptive neural cells via an Uptake_2_/PMAT process, and that intracellular dopamine remains chemically intact for sufficient lengths of time to modulate receptor-dependent intracellular signaling events. A functional role for intracellular D_5_ dopamine receptors was suggested by the results. This is probably the first demonstration of direct intracellular receptor-mediated signaling by dopamine in dopaminoceptive tissue, thus challenging the conventional notion that postsynaptic transmitter uptake serves merely as a mechanism for deactivating excess synaptic transmitter content. Given that CDP-diacylglycerol synthesis is rate-limiting for phosphatidylinositide neosynthesis or regeneration, our findings implicate a role for intracellular dopamine receptor coupling in the regulation of physiologic functions that are mediated through phospholipase C and phosphatidylinositol-3-kinase cascades at least in dopaminoceptive tissues. As illustrated in summary Figure 7, multiple gaps remain to be filled in order to better understand the sequence of events from transmembrane dopamine transport to intracellular modulation of CDP-diacylglycerol signaling. Further studies are needed to address these knowledge gaps and explore broader physiological consequences of pharmacologically modulating this multifunctional signaling nexus.

## Data Availability

The original contributions presented in the study are included in the article/Supplementary Material, further inquiries can be directed to the corresponding author.
