# Supplementary figures and images for: Dopamine internalization via Uptake2 and stimulation of intracellular D5-receptor-dependent calcium mobilization and CDP-diacylglycerol signaling
Source: Front Pharmacol. 2024 Oct 25;15:1422998. doi: 10.3389/fphar.2024.1422998 (PMC11543475; doi:10.3389/fphar.2024.1422998)

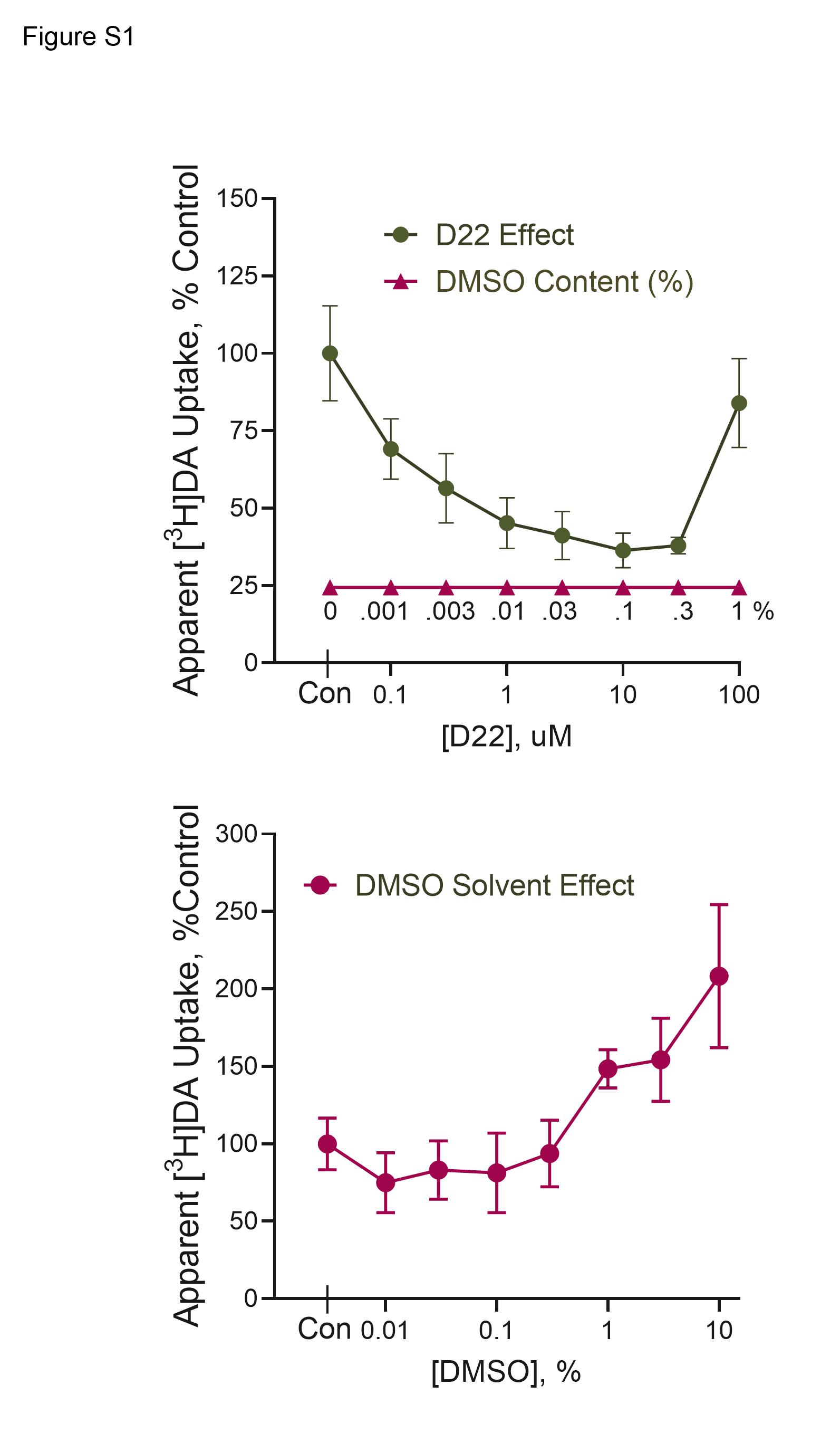

Supplement: Supplementary file 1 [file Image1.JPEG]
